# Anthocyanins, Carotenoids, and Betalains in Plants: From Biosynthesis to Function

**DOI:** 10.3390/plants15152303

**Published:** 2026-07-27

**Authors:** Farhat Abbas, Yanguo Ke, Yiwei Zhou, Tingting Yan, Huicong Wang, Fuchu Hu

**Affiliations:** 1 Institute of Tropical Fruit Trees, Hainan Academy of Agricultural Sciences/Key Laboratory of Genetic Resources Evaluation and Utilization of Tropical Fruits and Vegetables (Co-Construction by Ministry and Province), Ministry of Agriculture and Rural Affairs/Key Laboratory of Tropical Fruit Tree Biology of Hainan Province, Haikou 571100, China; 2 Yunnan Urban Agricultural Engineering & Technological Research Center, College of Economics and Management, Kunming University, Kunming 650208, China; 3 Guangdong Provincial Key Laboratory of Ornamental Plant Germplasm Innovation and Utilization, Environmental Horticulture Research Institute, Guangdong Academy of Agricultural Sciences, Guangzhou 510640, China; 4 Key Laboratory of Biology and Genetic Improvement of Horticultural Crops-South China/Guangdong Litchi Engineering Research Center, College of Horticulture, South China Agricultural University, Guangzhou 510640, China; 5 Sanya Research Institute, Hainan Academy of Agricultural Sciences, Sanya 572025, China

Plant specialized metabolites include a wide variety of bioactive substances that affect plant development, environmental acclimatization, and ecological relationships. Among these metabolites, anthocyanins, carotenoids, and betalains constitute three fundamental categories of plant pigments that determine plant colors, regulate stress resilience, and facilitate plant–environment interactions [1,2,3,4]. In addition to their esthetic and biological functions, these colors hold significant nutritional, medicinal, and industrial relevance. Despite extensive research, the intricate regulatory networks, adaptive strategies, and translational uses of plant pigments remain rapidly advancing areas of investigation. This document presents 12 original research and review articles of excellent quality that encompass a comprehensive study spectrum, from molecular biosynthesis and transcriptional regulation to physiological adaptation, ecological roles, and applications in agriculture and industry. This edition integrates multi-omics, molecular genetics, physiological phenotyping, and field agronomy methodologies, providing contemporary mechanistic insights and actionable solutions for the functional analysis and precision enhancement of plant pigment metabolites.

Anthocyanins are the predominant flavonoid pigments found in terrestrial flora, serving roles in photoprotection, antioxidant defense, pollinator appeal, and the enhancement of crop nutrition and agronomy [5,6,7]. The biosynthesis mechanism is regulated by structural enzymes and conserved MYB-bHLH-WD40 transcriptional regulatory modules, which are influenced by developmental and environmental cues [8]. This Special Issue covers seven research articles centered on anthocyanins, constituting its most extensive subject segment, addressing molecular regulatory mechanisms, phenotypic diversity, abiotic stress responses, and agronomic performance in both model plants and crop species. Carotenoids are vital isoprenoid pigments necessary for plant photosynthesis, photoprotection, and the manufacture of important phytohormones such as abscisic acid and strigolactones. The trio of articles concerning carotenoids in this edition broadens the traditional perspective on carotenoid roles, emphasizing stress-induced metabolic reconfiguration, chloroplast retrograde signaling, and the dual regulatory compromises between plant resilience to abiotic and biotic stresses, alongside their nutraceutical significance for humans [9,10]. Betalains are distinctive pigments generated from tyrosine, produced only by Caryophyllales species, and they demonstrate a thoroughly documented mutually exclusive accumulation pattern with anthocyanins in plants [9]. In contrast to anthocyanins and carotenoids, the regulation of betalain biosynthesis, its evolutionary adaptation, and its industrial applications are yet to be adequately investigated.

Lasmar et al. (Contribution 1) elucidated the function of ELONGATED HYPOCOTYL 5 (HY5) and recent developments in the COP1-centered regulation of anthocyanin biosynthesis, focusing on post-translational processes and COP1’s interaction with HY5. The authors emphasized that COP1 acts as a conserved repressive regulator of anthocyanin production in darkness across many plant species. Crucially, COP1 does not directly engage with multicomponent transcriptional complexes like the MYB-bHLH-WD40 (MBW) assembly; rather, its E3 ligase function targets specific protein substrates. Furthermore, COP1 facilitates the ubiquitination and degradation of specific MYB and bHLH elements, therefore indirectly influencing MBW-dependent transcription and other factors that affect pigment production. While the general repressive role of COP1 is widely maintained, the specific substrates and the structure of COP1-centric regulatory frameworks differ significantly across species, highlighting the interplay between a common regulatory principle and distinct species-specific variations in COP1-driven regulation.

Demmig-Adams et al. (Contribution 2) examined the function of zeaxanthin and other carotenoids in response to stress, particularly abiotic stress, and their implications for biotic defense. The authors provided an overview of the safeguarding properties of xanthophylls and the function of carotenoids as precursors to various plant stress hormones. Furthermore, the authors examine the biosynthetic routes of xanthophylls and carotenoid-derived plant stress hormones, the role of xanthophylls in the photoprotection of photosynthesis, the significance of carotenoids as vital human micronutrients, and the contributions of xanthophylls to membrane stability. Focus is placed on zeaxanthin’s role in both abiotic and biotic defense mechanisms, along with its influence on elements of the biotic defense system with differing targets. Several major loops in signaling cascades connect the chloroplast and nucleus, which are influenced by the chloroplast’s redox status and regulated by xanthophylls.

Zhang et al. (Contribution 3) elucidated the functional characteristics of dihydroflavonol 4-reductase (*DFR*) genes linked to anthocyanin production in blueberries (*Vaccinium corymbosum*). Investigators identified 36 *VcDFR* genes and organized them into five subfamilies. The genes *VcDFR4/7/11/29/30/33/34* were significantly expressed in blueberry fruits, notably at advanced ripening phases. Transient overexpression research using fruit substantiates the involvement of *VcDFR11/30* in anthocyanin production through the upregulation of structural genes associated with anthocyanin biosynthesis. Furthermore, VcMYB-1 and VcbHLHs transcription factors attach to the promoter region of *VcFDR11* and stimulate its expression. Similarly, Zeng et al. (Contribution 4) present a new light-responsive regulatory pathway in *Arabidopsis thaliana*, illustrating that the ABI4 transcription factor inhibits late biosynthesis genes of anthocyanin in a strictly HY5-dependent fashion. This research identifies a novel molecular connection among light signaling, seedling photomorphogenesis, chloroplast formation, and flavonoid metabolism, enhancing the upstream regulatory framework of light-triggered anthocyanin accumulation.

Joya-Dávila et al. (Contribution 5) perform a systematic assessment of the phenotypic diversity of anthocyanins and their agroecological significance in 10 indigenous Mexican maize genotypes and their hybrid offspring in natural rainfed agricultural settings. The study validates that purple totomoxtle (maize husk) coloration is a permanent, genotype-specific biocultural characteristic with uniform anthocyanin accumulation in both vegetative and reproductive tissues. Anthocyanin levels in totomoxtle rise by 30% at late maturity, but photosynthetic pigments reach their zenith in the early vegetative phases. Pigmented genotypes demonstrate enhanced grain quality, superior ear characteristics, and greater yield consistency, with the JCTM × LLMJ hybrid attaining a peak yield of 6.60 tons per hectare. This research confirms totomoxtle coloring as a dependable phenotypic indicator for the protection of local maize germplasm, agroecological robustness, and sustainable crop breeding.

Zykin et al. (Contribution 6) investigated rye (*Secale cereale* L.), notable among small grains for its extensive variation in kernel color, which is influenced by tissue-specific flavonoid synthesis. However, this characteristic is still inadequately explored from colorimetric, cytological, and biochemical viewpoints. We examined the distribution of anthocyanin and proanthocyanidin (PA) inside seed tissues of 26 rye lines with defined grain color genes, utilizing *CIE*LAB color assessment, GrainScan single-grain imaging, light microscopy, and MALDI-IMS tissue imaging. Violet rye stores cyanidins in the pericarp, but green rye has delphinidins in the aleurone; proanthocyanidins are present in the brown testa of all lines except for vi3 anthocyanin-deficient mutants. The *CIE*LAB hue angle h° consistently differentiates four unique grain color categories: yellow, green, brown, and violet.

Zhou et al. (Contribution 7) employed metabolomics and transcriptomics to examine the coloration of tepals in *Gloriosa superba* ‘Passion Flame’. Tepal coloration is influenced by the buildup of anthocyanins and the transcriptionally regulated breakdown of chlorophyll; Pg3G and isosalipurposide are the primary contributors to red and yellow flavonoid compounds, respectively. Essential pigment biosynthesis and chlorophyll breakdown genes demonstrate stage-specific variations in expression. Two MYB proteins (GsMYB75, GsMYB114) independently regulate the early and late stages of anthocyanin biosynthesis, with co-expressed bHLH, NAC, and AP2/ERF transcription factors also participating. These findings enhance understanding of floral pigment modulation in monocots and bolster molecular breeding in ornamental crops. Given that the research relies on historical omics data, subsequent biochemical assays and gene functional validation are essential to confirm the roles of candidate genes and elucidate pigmentation processes in Gloriosa and similar ornamental species.

Ao et al. (Contribution 8) addressed the molecular and metabolic underpinnings of yellow peel pigmentation in apples by utilizing three cultivars with varying peel colors: the yellow-skinned ‘Venus Gold’, the moderately red-skinned ‘Yanfu 8’, and the dark red-skinned ‘Red Love’. Using comprehensive transcriptome research, qRT-PCR confirmation, and metabolomic assessment of peel tissues, LAR1 was established as a crucial functional gene responsible for the yellow peel characteristic of ‘Venus Gold’. Metabolomic findings indicated that the increased concentration of catechin and epicatechin in yellow peel tissues is a vital metabolic contributor to the development of yellow pigmentation. Moreover, notable discrepancies in volatile metabolite compositions were observed among the three cultivars, suggesting a strong relationship between apple peel pigmentation and fruit scent attributes. This study identifies a new regulatory mechanism for the pigmentation of yellow-skinned peels, where increased LAR1 expression enhances the buildup of catechin and epicatechin. It also provides the first cohesive transcriptomic and metabolomic evidence to substantiate this pigmentation regulation system in apple peel.

Zhang et al. (Contribution 9) delineated TeMADS6, a MADS-box transcription factor that orchestrates the coordinated regulation of chlorophyll and carotenoid metabolism in marigold flower tissues. The constitutive overexpression of TeMADS6 led to a modified yellow-green petal phenotype compared to wild-type plants. Quantitative HPLC assessment revealed that transgenic petals showed considerable decreases in various carotenoid constituents, including lycopene, antheraxanthin, violaxanthin, zeaxanthin, and lutein, along with a notable increase in chlorophyll levels. Transcriptomic analysis additionally uncovered unique transcriptional alterations in florets overexpressing TeMADS6: core carotenoid biosynthetic genes TePSY1 and TeHYDB were markedly suppressed, whereas chlorophyll catabolism displayed gene-specific modulation, with TeNYC1 and TePPH2 significantly downregulated and TeSGR2 upregulated. These findings collectively suggest that TeMADS6 acts as a bifunctional transcriptional regulator that concurrently inhibits carotenoid production and influences chlorophyll breakdown to determine floral coloration. This research enhances mechanistic understanding of transcriptionally regulated pigment metabolic pathways during marigold flower development and offers significant genetic opportunities for molecular alteration of floral hue and lutein enhancement in marigold.

Timofeenko et al. (Contribution 10) studied the physiological, biochemical, and morphological reactions of two distinct lettuce cultivars (*Lactuca sativa* L.), ‘Gypsy’ and ‘Pomegranate Lace’, to blue light (BL), UV-B radiation, and their combinations under moderate baseline illumination. Plants were exposed to 48 h of low-intensity continuous UV-B under three lighting conditions: daytime white light (WL), daytime WL combined with blue light (BL), and independent overnight UV-B, to maintain UVR8 signaling without interference from visible light. For the crinkled, regionally colored ‘Pomegranate Lace’, the amalgamation of WL + BL + UV-B treatment enhanced chlorophyll and carotenoid levels, photosynthetic efficiency, and stomatal conductance, but respiration remained unchanged. These results suggest efficient communication between cryptochrome and UVR8 signaling, which maintains photochemical efficacy and enhances the formation of antioxidant phenolics and carotenoids. Conversely, the smooth-leaved, uniformly colored ‘Gypsy’ demonstrated increased stomatal conductance, augmented photosynthetic and respiratory rates, and superior PSII efficiency when exposed to UV-B and BL supplementation. Irrespective of the cultivar, spectral interventions diminished plant biomass and leaf area while enhancing total antioxidant capacity. BL and UV-B demonstrated more pronounced enhancing effects on antioxidant activity and PSII functionality in ‘Gypsy’, presumably owing to increased light penetration and diminished leaf reflectance. In general, prolonged exposure to low-intensity UV-B serves as a crucial spectrum regulator that variably influences photosynthetic and antioxidant mechanisms in lettuce. The combination of UV-B and blue light enhances photochemical resistance and metabolic resilience in a cultivar-specific approach.

Ke et al. (Contributions 11 and 12) performed multi-omics comparative analyses to clarify the molecular mechanisms that regulate flower colors and fragrance differences among various *Hydrangea arborescens* cultivars. Initially, an arrangement of the white-flowered ‘Annabelle’ (An) and the pink-flowered ‘Pink Annabelle’ (PA) demonstrated contrasting volatile compositions and metabolic oversight: GC–MS analysis revealed 25 and 21 floral volatile organic compounds (VOCs) in ‘An’ and ‘PA’, respectively, with common VOCs present in both cultivars; ‘An’ exhibited greater VOC diversity, whereas ‘PA’ contained elevated concentrations of benzenoid and phenylpropanoid volatiles contributing to a more pronounced floral aroma. UPLC–MS/MS metabolomics validated the enhanced phenylpropanoid production in ‘PA’, whereas transcriptome analysis revealed 11,653 differentially expressed genes (DEGs), including 7633 elevated genes associated with secondary metabolism. Key structural genes (*PAL*, *4CL*, *CHS*, *DFR*, *ANS*) and functional transcription factors (MYB, bHLH, ERF, WRKY) exhibited variable regulation among cultivars, with the flavonoid repressor AtMYB4 homolog being downregulated and the anthocyanin enhancer being upregulated. The AtMYB90 homolog is elevated in ‘PA’, along with a jasmonate-related AtWRKY75 homolog significantly activated in ‘PA’. Integrated omics data revealed that the synchronized transcriptional enhancement of phenylpropanoid and flavonoid pathways jointly enhances the color and fragrance of pink-flowered hydrangeas. In another study of three blue-hued hydrangea cultivars (‘DB’ deep blue, ‘LB’ light blue, ‘GB’ green blue), the amalgamation of anthocyanin metabolomics, transcriptomics, and *CIE*LAB L*a*b* color evaluation elucidated flower pigmentation processes. UPLC-MS/MS detected 47 flavonoid metabolites, predominantly consisting of six anthocyanin variants, with delphinidins comprising 90% of the overall anthocyanins in dark blue ‘DB’. A cumulative of 51 and 31 differentially expressed genes (DEGs) were identified in relation to anthocyanin, flavonoid, and chlorophyll biosynthesis, with pivotal pathway genes (CHS, CHI, F3H, F3′5′H, DFR, ANS) demonstrating peak expression in ‘DB’; notably, a significant DFR isoform was prominently expressed in ‘DB’ while they were downregulated in ‘LB’ and ‘GB’, aligning with cultivar-specific anthocyanin accumulation. The differential expression of numerous transcription factors, chiefly WRKY, ERF, bHLH, NAC, and AP2/ERF family members, strongly correlated with anthocyanin gene expression and the development of blue pigmentation. These results collectively elucidate the molecular regulatory mechanisms behind differences in hydrangea blossom color and fragrance, offering significant genetic resources for prospective ornamental hydrangea molecular breeding.

Although progress has been realized in this area, numerous critical problems still need to be confronted in subsequent research endeavors. First, the upstream signal-sensing and interference networks that connect developmental and environmental signals to pigment production deserve more thorough investigation. Furthermore, the collaborative and conflicting metabolic interactions involving anthocyanins, carotenoids, and betalains across tissues and developmental stages remain insufficiently clarified. Third, there is a need for comprehensive confirmation of the epigenetic and post-translational regulatory mechanisms that optimize pigment metabolism. Lastly, further research is needed into the ecological roles of plant pigments in multifaceted field settings, including plant-microbe interactions. Ultimately, precise genetic breeding and metabolic engineering methodologies should be refined to secure focused pigment enhancement without undermining plant development and yield outcomes.

We express sincere appreciation to all authors for their significant contributions of original research and review articles that comprise this comprehensive thematic Special Issue. We really appreciate the anonymous reviewers for their thorough assessment and insightful feedback that greatly enhanced the quality of all submissions. We express our gratitude to the editorial staff of *Plants* for their expert management and assistance during the submission, review, and publication phases. We expect this Special Issue to serve as a thorough and current resource for scholars in plant secondary metabolism, crop breeding, plant physiology, and natural product creation, while also motivating additional new research in plant pigment biology and its varied applications.

## References

[1] Proffit M., Lapeyre B., Buatois B., Deng X., Arnal P., Gouzerh F., Carrasco D., Hossaert-McKey M. (2020). Chemical signal is in the blend: Bases of plant-pollinator encounter in a highly specialized interaction. Sci. Rep..

[2] Nagegowda D.A., Gupta P. (2020). Advances in biosynthesis, regulation, and metabolic engineering of plant specialized terpenoids. Plant Sci..

[3] De Vega C., Herrera C.M., Dötterl S. (2014). Floral volatiles play a key role in specialized ant pollination. Perspect. Plant Ecol. Evol. Syst..

[4] Balcke G.U., Bennewitz S., Bergau N., Athmer B., Henning A., Majovsky P., Jiménez-Gómez J.M., Hoehenwarter W., Tissier A. (2017). Multi-omics of tomato glandular trichomes reveals distinct features of central carbon metabolism supporting high productivity of specialized metabolites. Plant Cell.

[5] Wang S., Li L.-X., Zhang Z., Fang Y., Li D., Chen X.-S., Feng S.-Q. (2022). Ethylene precisely regulates anthocyanin synthesis in apple via a module comprising MdEIL1, MdMYB1, and MdMYB17. Hortic. Res..

[6] Liu Y., Tikunov Y., Schouten R.E., Marcelis L.F., Visser R.G., Bovy A. (2018). Anthocyanin biosynthesis and degradation mechanisms in Solanaceous vegetables: A review. Front. Chem..

[7] Chen Z., Yuan J., Yao Y., Cao J., Yang W., Long Y., Liu J., Yang W. (2023). PhAAT1, encoding an anthocyanin acyltransferase, is transcriptionally regulated by PhAN2 in petunia. Physiol. Plant..

[8] Zhang P., Zhu H. (2023). Anthocyanins in Plant Food: Current Status, Genetic Modification, and Future Perspectives. Molecules.

[9] Tanaka Y., Sasaki N., Ohmiya A. (2008). Biosynthesis of plant pigments: Anthocyanins, betalains and carotenoids. Plant J..

[10] Rodriguez-Concepcion M., Avalos J., Bonet M.L., Boronat A., Gomez-Gomez L., Hornero-Mendez D., Limon M.C., Meléndez-Martínez A.J., Olmedilla-Alonso B., Palou A. (2018). A global perspective on carotenoids: Metabolism, biotechnology, and benefits for nutrition and health. Prog. Lipid Res..

